# Next-Generation Sequencing for the Diagnosis of Challenging Culture-Negative Endocarditis

**DOI:** 10.3389/fmed.2019.00203

**Published:** 2019-09-20

**Authors:** Manon Kolb, Vladimir Lazarevic, Stéphane Emonet, Alexandra Calmy, Myriam Girard, Nadia Gaïa, Yannick Charretier, Abdessalam Cherkaoui, Peter Keller, Christoph Huber, Jacques Schrenzel

**Affiliations:** ^1^Service of General Internal Medicine, Geneva University Hospitals (HUG), Geneva, Switzerland; ^2^Genomic Research Laboratory, Service of Infectious Diseases, Geneva University Hospitals, Geneva, Switzerland; ^3^Bacteriology Laboratory, Service of Laboratory Medicine, Geneva University Hospitals, Geneva, Switzerland; ^4^Service of Infectious Diseases, Geneva University Hospitals, Geneva, Switzerland; ^5^Institute of Medical Microbiology, University of Zurich, Zurich, Switzerland; ^6^Service of Cardiovascular Surgery, Geneva University Hospitals, Geneva, Switzerland

**Keywords:** next-generation sequencing, culture-negative endocarditis, clinical metagenomics, *Cardiobacterium hominis*, diagnosis

## Abstract

Diagnosis of culture-negative infective endocarditis usually implies indirect pathogen identification by serologic or molecular techniques. Clinical metagenomics, relying on next-generation sequencing (NGS) is an emerging approach that allows pathogen identification in challenging situations, as evidenced by a clinical case. We sequenced the DNA extracted from the surgically-removed frozen valve tissue from a patient with suspected infective endocarditis with negative blood and valve cultures. Mapping of the sequence reads against reference genomic sequences, a 16S rRNA gene database and clade-specific marker genes suggested an infection caused by *Cardiobacterium hominis*.

Pathogen identification is a cornerstone for the diagnosis of infective endocarditis (IE) and guides treatment duration and antibiotic choice. Yet, in up to 31% of patients with IE, cultures performed on blood and valve remain negative (1). Previous antibiotic treatment and fastidious growth requirements are main reasons for failure of microorganism identification (2). Bacteria of the HACEK group, intracellular germs or nutritionally variant streptococci require particular conditions for the growth in the laboratory, such as specific complex media or lysis-centrifugation blood culture system (2).

Broad-range PCR amplification which screens for bacterial pathogens using universal primers provides an adjunct in diagnosis of IE. Multimodal diagnostic assays, including serologies and specific real-time PCR amplification, further increase the diagnostic yield in blood-culture negative IE (3, 4).

Recently, metagenomic approach using high-throughput culture-independent techniques based on next-generation sequencing (NGS) has emerged and opened new perspectives for microbiological diagnosis in the clinical setting. Here, we report the identification of *Cardiobacterium hominis* by NGS in the vegetation of a culture negative IE, in which broad-range PCR amplification did not detect any microorganism.

In May 2018, a 58-year-old man, independent painter, consulted the emergency room complaining about breathlessness, chest tightness and chills for a couple of weeks. He had a good dental status with no history of recent dental procedure. Physical examination revealed signs of heart failure with no splenomegaly, peripheral embolic lesions, large-vessel emboli, cutaneous lesions, or retinal/conjunctival lesions. Transthoracic and then transesophageal echocardiographies showed a moderate to severe aortic regurgitation and a 15 × 8 cm mobile vegetation on the right leaflet. Laboratory tests showed leukocyte count 12.4 × 10^9^ cells/L (reference range 4–11 × 10^9^ cells/L), hemoglobin 130 g/L (reference range 140–180 g/L) and C-reactive protein at 87.3 mg/L (reference range 0–10 mg/L). Suspecting an infective endocarditis, empiric treatment was started with amoxicillin, amoxicillin/clavulanic acid and gentamicin (day 0). On day 3, an aortic valve replacement was performed, and a biological valve was placed without complications. Before pathogen identification, antibiotic treatment was already adjusted by probabilistic means to ceftriaxone, a third-generation cephalosporin. After detection of *C. hominis*, this regimen was pursued for a total duration of 6 weeks. The patient recovered well and went back to his former performance status.

Five sets of blood cultures, taken at 30 min intervals on day 0, i.e., before antibiotic treatment, remained negative on day 7. Culture of the excised valve and vegetation on standard enriched media came back negative. Results of indirect detection of infective agents were also negative. These included: (i) Specific PCR tests for *Chlamydophila pneumoniae, Mycoplasma pneumoniae*, and *Bordetella* spp. in oropharyngeal samples, *Treponema pallidum, Tropheryma whipplei, Brucella* spp., *Bartonella henselae*, and *Bordetella quintana* in the heart-valve and *Brucella* spp. in blood; (ii) *Legionella pneumophila* urinary antigen test, and (iii) serologies for *Coxiella burnetti, Bartonella henselae*, and *Brucella* spp.

The broad-range 16S rDNA PCR performed on a fragment of the aortic valve revealed negative. A portion of the valve was further analyzed by clinical metagenomics (Supplementary Methods). DNA was extracted from two fragments (S1 and S2) of the valve specimen using a procedure that removes a significant amount of human DNA in order to allow deeper assessment of the bacterial component by NGS (5–8). One of the two fragments (S2) was mechanically grinded prior to DNA extraction. DNA quantification by qPCR revealed higher bacterial and lower human DNA concentrations in the mechanically pre-treated valve fragment as compared to the sample without this pre-treatment (Table 1). To identify bacterial species in the valve specimen, we performed NGS of both DNA extracts and analyzed sequence data by classifying sequence reads against: (i) reference genomic sequences using CLARK (9); (ii) clade-specific marker genes with MetaPhlAn2 (10), and (iii) curated 16S rRNA gene database (11) with mothur (12) and UBLAST (13). The results obtained by either approach suggested an infection caused by *Cardiobacterium hominis* (Figure 1; Supplementary Table 1). The reads assigned to this species were by far the most abundant (>97%) among the bacterial reads, as identified by CLARK. Similarly, most (>99.2%) reads that mapped to the 16S rRNA genes belonged to genus *Cardiobacterium*. *Cardiobacterium* was the only genus identified by mapping of reads against clade-specific markers, with a dominance of *C*. *hominis* for both DNA extracts. *Cardiobacterium* was not identified by sequencing of negative extraction controls (NEC1 and NEC2) by any of the three analysis methods used.

**Table 1:** Load of bacterial and human DNA in DNA extracts of the heart valve and in negative controls determined by qPCR and NGS.

**Sample^a^**	**DNA concentration (pg/uL)^b^**			**NGS reads**			
	**Human**	**Bacterial**	**% Bacterial^c^**	**Raw**	**Filtered non-human^d^**	**Assigned to prokaryotes**	**% Prokaryotic^e^**
S1	16,355	140	0.84	1,491,700	2,603	2,437	0.81
S2	219	718	76.63	1,396,937	192,031	184,618	81.14
NEC1	0	0.19	100	24,813	4,438	3,677	94.4
NEC2	0	1.31	100	83,731	7,494	5,141	54.4

a Aortic samples S1 and S2 and negative extraction controls (NEC1 and NEC2).

b Determined by qPCR tests.

c Relative to the sum of bacterial and human DNA.

d After removal of low-quality, human and replicate reads.

e The percentage was determined relative to the sum of (de-replicated) reads assigned with CLARK to Homo sapiens and prokaryotic phyla.

**Figure 1:**
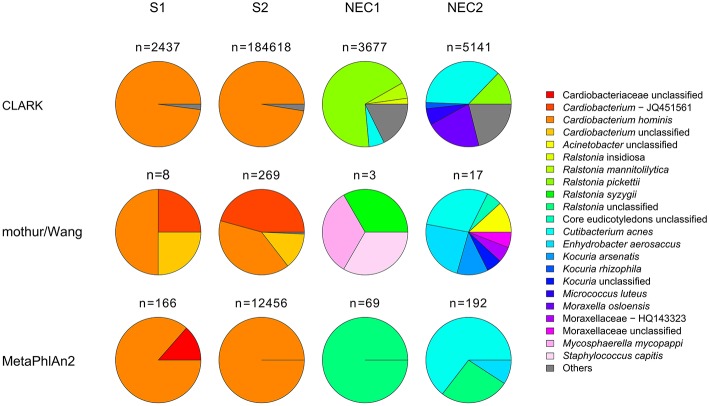
Relative abundance of prokaryotic taxa determined by classification of genome fragment sequences (CLARK), classification of 16S rRNA gene fragments (mothur/Wang) and read mapping to clade markers (MetaPhlAn2). All taxa with relative abundance <1% were summed up and represented as “others.” For simplicity, in the mothur/Wang approach, only results obtained with forward sequence reads are presented. *n*, number of hits obtained for each sample and method used.

Herein, we present the case of a culture-negative IE in which the all-round standard multimodal diagnostic work-up, aimed at identifying causative agents, came back negative. The final diagnosis was achieved by NGS of the aortic native valve DNA extract, which identified *C. hominis*, a member of the HACEK group that accounts for 1.2–3% of IE cases (14). Only 61 cases of *C. hominis* IE have been reported in the English-language literature between 1962 and 2005 (15).

*C. hominis* is a facultatively anaerobic, slow-growing, fastidious, non-motile, gram-negative rod of low virulence, commonly present in normal oral and nasal human microbiota. It is the third most common agent responsible for HACEK endocarditis (14). Failure to identify *C. hominis* in blood cultures, which were taken before antibiotic treatment of our patient, may be ascribed to the fastidious nature of the organism.

In previous studies, testing heart valves for bacterial pathogens in patients with suspected IE revealed higher specificity of broad-range PCR as compared to standard cultures, even though some patients with positive cultures tested negative by PCR (16, 17). Various reasons have been attributed to false negative broad-range PCR results: low bacterial load due to the time lapsing between antibiotic therapy and surgical intervention, use of a non-representative valve fragment (with low local bacterial density), and PCR inhibition (17) which may depend on DNA extraction procedure and PCR conditions. In our case, antibiotic treatment could be a possible cause of the culture-negative results of the valve tissue but unlikely to have caused failure in pathogen detection by PCR, because bacterial DNA load (which may originate from either live or dead cells) in the extracts of valve fragments used for NGS was relatively high (>2 × 10^6^ and >10^7^ rRNA gene copies for S1 and S2, respectively; Table 1). It is more likely that the broad-range PCR detection was compromised by the presence of PCR inhibitors. Yet, a sampling bias cannot be ruled out, since broad-range PCR and NGS assessed different specimen fragments. In addition, it has been recently shown that specific steps in sample preparation of the valve specimen may affect the diagnostic yield (18).

In a previous study, Imai et al. found good correlation between NGS and culture data obtained on the native valve from two patients with blood culture-positive endocarditis (19). Two other cases of blood culture-negative endocarditis were reported where the pathogen could be identified by NGS (19, 20). In our case, the identification of the causative agent of IE by NGS was reinforced by the use of three different bioinformatics tools relying on different reference databases. We believe this point is of crucial importance and guarantees for a curated and redundant signal and therefore a more reliable diagnosis.

Currently, the application of NGS approach in routine diagnostic testing is relatively costly unless larger batches of samples are processed. The application of NGS to various samples is of increasing clinical interest because it allows faster pathogen identification than standard culture. A complete metagenomics analysis of a clinical sample is now possible in <30 h using Illumina sequencing platforms. This turn-around time is longer than that of PCR and qPCR assays; however, it circumvents the need for multiple assays, e.g. target-specific PCRs aimed at confirming broad range PCR results (21), and has the potential to (i) detect multiple organisms (including uncommon pathogens), (ii) perform bacterial typing and (iii) predict antibiotic resistance profile (6, 22), all in the same test. Mechanical pre-treatment of valve tissue followed by the removal of DNA from selectively lysed human cells seem to increase the likelihood of pathogen identification. NGS of cell-free circulating plasma or serum DNA is another promising diagnostic tool for the detection of bacteremia in patients with IE (23). Our case clearly demonstrates that there is a lack in conventional techniques and that there is a clinical benefit to perform NGS in selected situations such as culture- and PCR-negative results. In particular the implementation of multiple analysis approaches and the use of orthogonal assessment to validate the obtained results should be promoted further.

## Data Availability

The datasets generated for this study can be found in European Nucleotide Archive (ENA), PRJEB25228.

## Ethics Statement

Written informed consent has been obtained from the patient for the publication of this case report.

## Author Contributions

MK, VL, SE, ACa, ACh, CH, and JS analyzed and interpreted patient data. MG, NG, PK, and ACh performed the experiments. NG and VL analyzed the metagenomics data. MK, VL, YC, and JS wrote the manuscript. All authors read and approved the final manuscript.

### Conflict of Interest Statement

The authors declare that the research was conducted in the absence of any commercial or financial relationships that could be construed as a potential conflict of interest.
