# Supplementary material for: Next-Generation Sequencing for the Diagnosis of Challenging Culture-Negative Endocarditis
Source: Front Med (Lausanne). 2019 Sep 20;6:203. doi: 10.3389/fmed.2019.00203 (PMC6763761; doi:10.3389/fmed.2019.00203)
Supplement: Supplementary file 1 [file Data_Sheet_1.docx]

**Supplementary Table 1. Mapping of metagenomic reads to reference 16S rRNA gene sequences.** Reads corresponding to 16S rRNA genes were extracted from the NGS dataset and mapped to reference sequences using UBLAST with a minimum identity of 95%. The results were considered unique or multiple, depending on whether a single or multiple best hits were found. ‘Total’ represents the sum of reads with unique and multiple best hits. The best-hit counts were stratified by identity percentages with an increment of 1, starting from 95%. Empty cells correspond to 0 counts.

| **Sample** | **Number of hits** | **Reference  OTU Number** | **OTU Taxonomy** | **Percent identity** | | | | |
| --- | --- | --- | --- | --- | --- | --- | --- | --- |
|  |  |  |  | **95-96** | **96-97** | **97-98** | **98-99** | **99-100** |
| **S1** | **Unique/Total** 6/8 | 85897 | Cardiobacterium hominis |  |  |  |  | 6 |
|  | **Multiple/Total** 2/8 | 135952 | Cardiobacterium JQ451561 |  |  |  |  | 2 |
|  |  | 135961 | Cardiobacterium JQ455380 |  |  |  |  | 1 |
|  |  | 135967 | Cardiobacterium JQ455990 |  |  |  |  | 1 |
|  |  | 85897 | Cardiobacterium_hominis |  |  |  |  | 2 |
| **S2** | **Unique/Total** 124/269 | 85897 | Cardiobacterium_hominis |  |  |  | 2 | 122 |
|  | **Multiple/Total** 145/269 | 134213 | Cardiobacterium JN713341 |  |  |  |  | 1 |
|  |  | 134229 | Cardiobacterium JN713405 |  |  |  |  | 1 |
|  |  | 135952 | Cardiobacterium JQ451561 |  |  |  | 1 | 126 |
|  |  | 135961 | Cardiobacterium JQ455380 |  |  |  |  | 14 |
|  |  | 135967 | Cardiobacterium JQ455990 |  |  |  | 1 | 80 |
|  |  | 85897 | Cardiobacterium_hominis |  |  |  | 1 | 144 |
|  |  | 87912 | Cardiobacterium_valvarum |  |  |  |  | 3 |
| **NEC1** | **Unique/Total** 1/3 | 142668 | Mycosphaerella mycopappi | 1 |  |  |  |  |
|  | **Multiple/Total** 2/3 | 101216 | Ralstonia solanacearum |  |  |  |  | 1 |
|  |  | 135001 | Ralstonia pickettii |  |  |  |  | 1 |
|  |  | 139860 | Ralstonia syzygii |  |  |  |  | 1 |
|  |  | 139861 | Ralstonia syzygii |  |  |  |  | 1 |
|  |  | 141149 | Staphylococcus saccharolyticus |  |  |  |  | 1 |
|  |  | 141151 | Staphylococcus epidermidis |  |  |  |  | 1 |
|  |  | 142622 | Ralstonia syzygii |  |  |  |  | 1 |
|  |  | 80683 | Staphylococcus caprae |  |  |  |  | 1 |
|  |  | 82549 | Staphylococcus capitis |  |  |  |  | 1 |
|  |  | 87879 | Ralstonia insidiosa |  |  |  |  | 1 |
| **NEC2** | **Unique/Total** 3/17 | 143566 | Kocuria rhizophila |  |  |  |  | 1 |
|  |  | 91721 | Acinetobacter towneri |  |  |  |  | 1 |
|  |  | 96890 | Moraxella osloensis |  |  |  |  | 1 |
|  | **Multiple/Total** 14/17 | 100880 | Citrus sinensis |  |  |  |  | 1 |
|  |  | 100955 | Daucus carota |  |  |  |  | 1 |
|  |  | 102120 | Mangifera indica |  |  |  |  | 1 |
|  |  | 102123 | Cercidiphyllum japonicum |  |  |  |  | 1 |
|  |  | 102124 | Hamamelis japonica |  |  |  |  | 1 |
|  |  | 102125 | Heuchera micrantha |  |  |  |  | 1 |
|  |  | 102126 | Itea virginica |  |  |  |  | 1 |
|  |  | 102132 | Pterostemon rotundifolius |  |  |  |  | 1 |
|  |  | 102135 | Saxifraga stolonifera |  |  |  |  | 1 |
|  |  | 102229 | Buxus microphylla |  |  |  |  | 1 |
|  |  | 103376 | Panax ginseng |  |  |  |  | 1 |
|  |  | 104318 | Anethum graveolens |  |  |  |  | 1 |
|  |  | 104709 | Ipomoea purpurea |  |  |  |  | 1 |
|  |  | 105478 | Cuscuta exaltata |  |  |  |  | 1 |
|  |  | 109501 | Guizotia abyssinica |  |  |  |  | 1 |
|  |  | 121877 | Acinetobacter GU356270 |  |  |  |  | 1 |
|  |  | 122359 | Acinetobacter GU968454 |  |  |  |  | 1 |
|  |  | 123357 | Acinetobacter HM126908 |  |  |  |  | 1 |
|  |  | 125278 | Acinetobacter HQ143323 |  |  |  |  | 2 |
|  |  | 128565 | Propionibacterium JF096764 |  |  |  |  | 1 |
|  |  | 128888 | Propionibacterium JF180432 |  |  |  |  | 1 |
|  |  | 134019 | Acinetobacter puyangensis |  |  |  |  | 1 |
|  |  | 140358 | Acinetobacter indicus |  |  |  |  | 1 |
|  |  | 140756 | Kocuria arsenatis |  |  |  |  | 2 |
|  |  | 140990 | Acinetobacter albensis |  |  |  |  | 1 |
|  |  | 141829 | Nicotiana plumbaginifolia |  |  |  |  | 1 |
|  |  | 143566 | Kocuria rhizophila |  |  |  |  | 2 |
|  |  | 143654 | Nicotiana tabacum |  |  |  |  | 1 |
|  |  | 82733 | Nicotiana sylvestris |  |  |  |  | 1 |
|  |  | 82753 | Nicotiana tomentosiformis |  |  |  |  | 1 |
|  |  | 85613 | Propionibacterium acnes |  |  |  |  | 2 |
|  |  | 88095 | Propionibacterium humerusii |  |  |  |  | 1 |
|  |  | 88137 | Propionibacterium AFUN |  |  |  |  | 2 |
|  |  | 88868 | Atropa belladonna |  |  |  |  | 1 |
|  |  | 89315 | Enhydrobacter aerosaccus |  |  |  |  | 4 |
|  |  | 90089 | Solanum lycopersicum |  |  |  |  | 1 |
|  |  | 90696 | Cuscuta reflexa |  |  |  |  | 1 |
|  |  | 91598 | Gossypium barbadense |  |  |  |  | 1 |
|  |  | 91716 | Acinetobacter gerneri |  |  |  |  | 1 |
|  |  | 91719 | Acinetobacter soli |  |  |  |  | 1 |
|  |  | 91962 | Acinetobacter rudis |  |  |  |  | 1 |
|  |  | 92201 | Propionibacterium acnes |  |  |  |  | 5 |
|  |  | 93779 | Panax ginseng |  |  |  |  | 1 |
|  |  | 94288 | Eucalyptus globulus |  |  |  |  | 1 |
|  |  | 95608 | Vitis vinifera |  |  |  |  | 1 |
|  |  | 96890 | Moraxella osloensis |  |  |  |  | 4 |
|  |  | 98292 | Solanum tuberosum |  |  |  |  | 1 |
|  |  | 98751 | Gossypium hirsutum |  |  |  |  | 1 |
|  |  | 98773 | Solanum bulbocastanum |  |  |  |  | 1 |
|  |  | 98774 | Solanum lycopersicum |  |  |  |  | 1 |
|  |  | 98942 | Helianthus annuus |  |  |  |  | 1 |
|  |  | 98944 | Solanum tuberosum |  |  |  |  | 1 |
|  |  | 99945 | Buxus sempervirens |  |  |  |  | 1 |

**Supplementary Methods**

***DNA extraction***. Frozen cardiac valve tissue was cut with a scalpel into two pieces of 84 mg (fragment S1) and 97 mg (fragment S2), respectively, on a disposable Petri dish. Fragment S1 was shredded with a scalpel. S2 was partly homogenized with an Omni Hard Tissue Homogenizing Mix (2-mL tubes) (Tulsa, Oklahoma, US) on a Bead Ruptor 4 (Omni) at speed 4 for 30 seconds. DNA was extracted from both fragments using the Ultra-Deep Microbiome Prep kit (Molzym, Bremen, Germany) according to the manufacturer’s instructions, eluted in 100 µL and stored at –20°C. Negative extraction controls for both DNA extraction protocols (NEC1 and NEC2) were performed by using 100 µL of the PKB Buffer (Molzym) instead of clinical sample.

***qPCR assays.*** The concentrations of human and bacterial DNA were determined by qPCR experiments as described previously (1), targeting beta-actin and 16S rRNA reference genes, respectively. For easier comparison to human DNA load, we converted the 16S rRNA gene copy number to DNA mass by considering that 1 pg DNA corresponds to 1,493 copies of the 16S rRNA gene (figures valid for *Escherichia coli* genomic DNA used to make a reference curve).

***DNA sequencing.*** Metagenomic libraries were prepared from 1 ng DNA (sum of bacterial and human DNA load determined by qPCR) for clinical samples, and 5 µL DNA extract for negative extraction controls (1 pg and 6.5 pg DNA for NEC1 and NEC2, respectively) using Nextera XT DNA Sample Preparation Kit (Illumina, San Diego, USA). The libraries were sequenced (2 × 250) at the Genomics Platform of the University of Geneva on an Illumina MiSeq instrument using the MiSeq Reagent Kit v2.

***Bioinformatics analysis.*** We used Trimmomatic v.0.36 package (2) to: (i) remove bases corresponding to the standard Illumina adapters; (ii) to cut bases off the start or the end of a read, if below quality threshold of 5, and (iii) to trim low-quality ends of reads at the beginning of any 20-base sliding window with an average Phred quality <30. The reads that after trimming step had a length <150 bases were discarded.

Reads matching the human genomic sequence were identified using CLARK (3) v.1.2.3.2 with the default parameters and GRCh38.p7 database (4, 5). After removal of the reads classified at the phylum level to Chordata (to which belongs the species *Homo sapiens*), the data were deposited to European Nucleotide Archive (ENA) database under study number PRJEB25228.

To filter out putative artificial replicate reads, we used a homemade script that retains the longest sequence from those with identical first 100 bases, in either forward or reverse reads. Any forward or reverse reads without its corresponding paired read were discarded. Remaining reads were classified at the species and phylum taxonomic levels using CLARK (with parameters -m 0 -c 0.8) against the collection of complete prokaryotic and viral genomes from the NCBI RefSeq (5) database (downloaded on October 14, 2017), containing, in addition, available unfinished genome sequences of *C*. *hominis* and *C*. *valvarum* strains. The percentage of each prokaryotic species was determined relative to the total number of prokaryotic reads identified at the phylum level.

Sequencing reads were mapped to bacterial/archaeal 16S rRNA genes using USEARCH (6) v.8.1.1861 (-usearch_local -id 0.9 -query_cov 1 -top_hit_only -strand both) against the EzBioCloud 16S database (7) (downloaded on September 5, 2017). The selected reads were classified via mothur’s v.1.39.5 (8) command classify.seq (-method wang -cutoff 80) based on the Wang (9) approach using the EzBioCloud 16S database. They were also compared against the EzBioCloud 16S database using UBLAST from the USEARCH package (-ublast -id 0.95 -evalue 0.00001 -strand both -top_hits_only) and stratified by the percentage of identity in the 95–100% range with an increment of 1%. We discriminated the reads with single from those with multiple best hits.

MetaPhlAn2 (10) taxonomic profiling, based on read mapping against clade markers, was used with default settings.

**References**

1. Lazarevic V, Gaïa N, Emonet S, Girard M, Renzi G, Despres L, et al. Challenges in the culture-independent analysis of oral and respiratory samples from intubated patients. *Front Cell Infect Microbiol*. (2014) 4:65.

2. Bolger AM, Lohse M, Usadel B. Trimmomatic: a flexible trimmer for Illumina sequence data. *Bioinformatics*. (2014) 30:2114-20.

3. Ounit R, Wanamaker S, Close TJ, Lonardi S. CLARK: fast and accurate classification of metagenomic and genomic sequences using discriminative k-mers. *BMC Genomics*. (2015) 16:236.

4. International Human Genome Sequencing C. Initial sequencing and analysis of the human genome. *Nature*. (2001) 409:860-921.

5. Pruitt KD, Tatusova T, Maglott DR. NCBI reference sequences (RefSeq): a curated non-redundant sequence database of genomes, transcripts and proteins. *Nucleic Acids Res*. (2007);35(Database issue) D61-5.

6. Edgar RC. Search and clustering orders of magnitude faster than BLAST. *Bioinformatics*. (2010) 26:2460-1.

7. Yoon S-H, Ha S-M, Kwon S, Lim J, Kim Y, Seo H, et al. Introducing EzBioCloud: a taxonomically united database of 16S rRNA gene sequences and whole-genome assemblies. *Int J Syst Evol Microbiol*. (2017) 67:1613-7.

8. Schloss PD, Westcott SL, Ryabin T, Hall JR, Hartmann M, Hollister EB, et al. Introducing mothur: open-source, platform-independent, community-supported software for describing and comparing microbial communities. *Appl Environ Microbiol*. (2009) 75:7537-41.

9. Wang Q, Garrity GM, Tiedje JM, Cole JR. Naive Bayesian classifier for rapid assignment of rRNA sequences into the new bacterial taxonomy. *Appl Environ Microbiol*. (2007) 73:5261-7.

10. Truong DT, Franzosa EA, Tickle TL, Scholz M, Weingart G, Pasolli E, et al. MetaPhlAn2 for enhanced metagenomic taxonomic profiling. *Nat Meth*. (2015) 12:902-3.
